# Oral Bisphosphonates and Upper Gastrointestinal Cancer Risks in Asians with Osteoporosis: A Nested Case-Control Study Using National Retrospective Cohort Sample Data from Korea

**DOI:** 10.1371/journal.pone.0150531

**Published:** 2016-03-03

**Authors:** Sun-Young Jung, Hyun Soon Sohn, Eun-Ja Park, Hae Sun Suh, Ji-Won Park, Jin-Won Kwon

**Affiliations:** 1 Office of Pharmacoepidemiology, Korea Institute of Drug Safety and Risk Management, Anyang, Gyeonggi-do, Korea; 2 Graduate School of Clinical Pharmacy, CHA University, Gyeonggi-do, Korea; 3 Korea Institute for Health and Social Affairs, Sejong, Korea; 4 College of Pharmacy, Pusan National University, Busan, Korea; 5 College of Natural Science, Kyungpook National University, Daegu, Korea; 6 College of Pharmacy and Research Institute of Pharmaceutical Sciences, Kyungpook National University, Daegu, Korea; College of Medicine, National Taiwan University, TAIWAN

## Abstract

**Background:**

Bisphosphonate can irritate the gastrointestinal mucosa and increase the risk of esophageal or gastric cancer. The relatively high prevalence of upper gastrointestinal cancers and the widespread use of bisphosphonate in Korea call for further investigation. We conducted a case-control study to evaluate the risk of esophageal or gastric cancer after exposure to oral bisphosphonates in Korean patients with osteoporosis.

**Methods:**

We used the National Health Insurance Service-National Sample Cohort database of Korea from 2002 to 2013. Among osteoporotic patients (>40 years), cases were defined as incident diagnosis of esophageal or gastric cancer between 2006 and 2013. For each case, four controls were matched for age, sex, and income level by type of insurance. We categorized bisphosphonate use as non-user, recent user, past user, and past and recent user, depending on prescription in two periods (1 to 2 years and 2 to 4 years prior to the index date). We also assessed the duration of bisphosphonate use by measuring cumulative duration of exposure (CDE). To examine the association between oral bisphosphonates and esophageal or gastric cancer, we estimated adjusted odds ratios (aORs) and 95% confidence intervals (CIs) using conditional logistic regression analysis, adjusting for concomitant diseases.

**Results:**

A total of 1,708 cases and 6,832 controls were identified. The aORs (95% CIs) of recent, past, and continuous bisphosphonate use compared to non-users were 1.18 (0.93–1.51), 1.04 (0.83–1.29), and 1.25 (0.95–1.58)), respectively. In addition, no significant association was observed by CDE, even when different outcome definitions were applied.

**Conclusions:**

This study did not prove an increased risk of esophageal or gastric cancer risk associated with bisphosphonate use, with respect to both risk windows and duration of exposure, in an Asian population-based, real-world setting.

## Introduction

The rapid increase in the elderly population leads to concern about the increasing prevalence of osteoporosis as a common senile disease. This is serious particularly in an aging society such as South Korea, where people over 65-years of age account for more than 13% of the total population[1]. Asian women are at the highest risk for osteoporosis because of differences in bone mass and density as well as ethnic differences[2, 3]. A recent study reported that 19.3% of Koreans aged ≥50 years are estimated to have osteoporosis[4].

Clinical guidelines recommend the treatment of osteoporosis to decrease fracture risks[5, 6], because it may result in national healthcare budget savings[7]. Bisphosphonates are widely prescribed, worldwide, as a primary drug for the purposes of osteoporosis treatment[8]. However, the increased use of bisphosphonates raises safety issues while having the desired effect of fracture prevention[9, 10]. It has emerged that use of oral bisphosphonate formulations (especially nitrogen-containing alendronate) could lead to adverse events such as upper gastrointestinal (GI) cancers, including those of the esophagus[11] and stomach[12]. However, while some studies suggested a significant association between bisphosphonates and upper GI cancers[13], others reported different results[14, 15].

Until now, meta-analyses of observational studies have found no significant association between bisphosphonates and esophageal or gastric cancer, but further, well-designed studies and analyses on dose-response and the duration of the treatment time are warranted[16–18]. Moreover, these previous studies were primarily located in Europe (including the United Kingdom [UK]) and the United States (US). An Asian study, conducted in Taiwan, recently reported increased risks of overall cancer incidence at higher doses of alendronate; however, no significant relationship with esophageal or gastric cancers were identified due to the small numbers of cases and a lack of statistical power[19].

Bisphosphonates account for over 80% of the total osteoporosis treatment drug market in Korea, and the market is steadily increasing[20, 21]. In addition, considering the relatively high prevalence of gastric cancer in the Korean population[22], a population-based study is necessary for seeking ways for appropriate bisphosphonate use. This study was conducted to determine the association between oral bisphosphonate treatment in patients with osteoporosis and the risk of upper gastrointestinal cancers (esophageal and gastric cancer) in a nationwide cohort of Korea.

## Materials and Methods

### Data source

We used the Korean National Health Insurance Service-National Sample Cohort (NHIS-NSC) Database from 2002 to 2013 [23]. A total of 1 million subjects from the 756 strata using three kinds of variables (age [18 groups], sex [2 groups], and income level according to type of insurance [10 groups for both NHI district subscriber and NHI employee subscriber, and one group as medical aid: total of 21 groups]) were randomly selected from the NHIS-NSC database in 2002 and followed up until 2013.

The NHIS-NSC database comprises a semi-dynamic cohort; data regarding newborns are added to the database every year to supplement the loss of numbers due to deaths. The NHIS-NSC database was validated by its representativeness from the overall Korean population[24]. The database includes data on subject demographics; clinical information, such as disease diagnosis, drug prescription, and healthcare costs; beneficiary’s social economic level; and death records. Disease diagnoses held in the database are coded based on the International Codes of Disease 10^th^ Edition Clinical Modification (ICD-10-CM). This study was approved by the Institutional Review Board by Kyungpook National University (KNU 2014–57). Informed consent was not obtained because patient records/information was anonymized and de-identified prior to analysis.

### Data availability statement

This database is not publicly available, and its use was restricted to users who gain approval for access by NHIS. We applied for the data access to NHIS together with the study protocol which got an approval from Institutional Review Board of principal investigator’s affiliation, and got an approval from NHIS.

### Study population and design

In this nested case-control study, the cohort population was defined as subjects with the primary- and sub-diagnosis codes regarding osteoporosis (ICD-10 codes: M80 –osteoporosis with pathological fracture; M81 –osteoporosis without pathological fracture; and M82 –osteoporosis in disease classified elsewhere) were identified during 2002 and 2013. The patients with related gastric tract cancers (ICD-10 codes: C17-C26), except for esophageal or gastric cancer (ICD-10 codes: C15 –malignant neoplasm of esophagus and C16 –malignant neoplasm of stomach), were excluded from the cohort population.

The cases were defined as having their first diagnosis of esophageal or gastric cancer (ICD-10 codes: C15 and C16) from 2006 to 2013. The index date was defined as the date of the first diagnosed esophageal or gastric cancer. Patients with previous diagnoses of esophageal or gastric cancer from 2002 to 2005 were excluded.

The control group was established by matching with each case for gender (male/female), age (5 year interval), and income level (10 categories) by the type of insurance (NHI district subscriber, NHI employee subscriber, and medical aid). For matching, the greedy method was used to identify these controls[25]. When the case was selected, the nearest neighbor control was lined up. For 1:4 matching, the closest four controls were selected. The first selected control was not replaced.

### Exposure to bisphosphonate

Bisphosphonates (alendronate, risedronate, etidronate, clodronate, ibandronic acid, and pamidronate) were identified via the prescription records in the NHIS-NSC database using the Anatomical Therapeutic Chemical (ATC) classification system of the World Health Organization (WHO). All prescriptions for the bisphosphonates without missing of any prescriptions were included. To investigate the effect of gastrointestinal exposure to bisphosphonate, only oral forms of the drugs were considered in base case analysis. The duration of bisphosphonate exposure was calculated by the consideration of the formulation such as sustained release of one year, one month, one week, or one day. The exposure of bisphosphonate was determined by the initiation and continuation of bisphosphonate prescription during the four years prior to the index date. However, one year (0–1 year) just before the index date was excluded to minimize bias because this exposure window could be non-relevant exposure period to investigate association of drug-cancer in consideration of induction and latency point of view[12, 26].

The observational period was divided into: Period A (2 to 4 years prior to the index date) and Period B (1 to 2 years prior to the index date). A patient who took at least one prescription during the observational period was considered to be a bisphosphonate user. The study cohort was classified into four mutually exclusive groups according to their exposure to bisphosphonate during these observational periods: non-user, recent user, past user, and past and recent user. A non-user was defined as having no records of a bisphosphonate prescription at any time during Period A or B. A recent user was defined as having no exposure to bisphosphonate during Period A and exposure in Period B. A past user was defined as having exposure to bisphosphonate during Period A and no exposure during Period B. Finally, a past and recent user was defined as having exposure to biphosphonate in both Period A and B.

The association between bisphosphonate exposure level and the upper gastrointestinal cancer (i.e. esophageal or gastric cancer) was investigated by cumulative duration of exposure (CDE). CDE was defined as the percentage of total prescription days of bisphosphonates during the overall observation periods. The bisphosphonate exposed proportion of the study cohort was further classified according to four CDE groups: 0%<CDE<25%, 25%≤CDE<50%, 50%≤CDE<75%, and 75%≤CDE≤100%.

### Sensitivity analysis

We conducted several sensitivity analyses to investigate the robustness of the study results. First, the exposure of bisphosphonate was expanded to include both oral or injection dosage forms. Second, the definition of cases were defined as follows: 1) esophageal cancer diagnosis only, 2) gastric cancer diagnosis only, 3) esophageal or gastric cancer with hospitalization during the first year after the index date, and 4) diagnosis of esophageal or gastric cancer with anticancer agent usage or surgical operation (curative operation of esophageal malignant tumor [Q2401-3]; total gastrectomy [QA536, Q2533-4, and Q2536-7]; subtotal gastrectomy [Q0251-9, Q2594, and Q2598]) from 2006 to 2013. Third, all related gastrointestinal tract cancers (ICD-10 codes C17-C26) which were not esophageal or gastric cancer, were not excluded from the cohort population.

### Statistical analysis

The demographic and clinical information between cases and controls was summarized by descriptive statistics. Categorical variables were summarized by frequency and continuous variables were summarized by mean and standard deviation. We performed a conditional logistic regression analysis to calculate the odds ratios (ORs) and 95% confidence intervals (CIs) to quantify the association between bisphosphonate exposure and esophageal or gastric cancer. We adjusted for concomitant disease, such as diabetes and rheumatoid arthritis, based on ICD-10 code. This study complied with STROBE (Strengthening the Reporting of Observational Studies in Epidemiology) guidelines for case-control studies[27].

## Results

**Fig 1:**
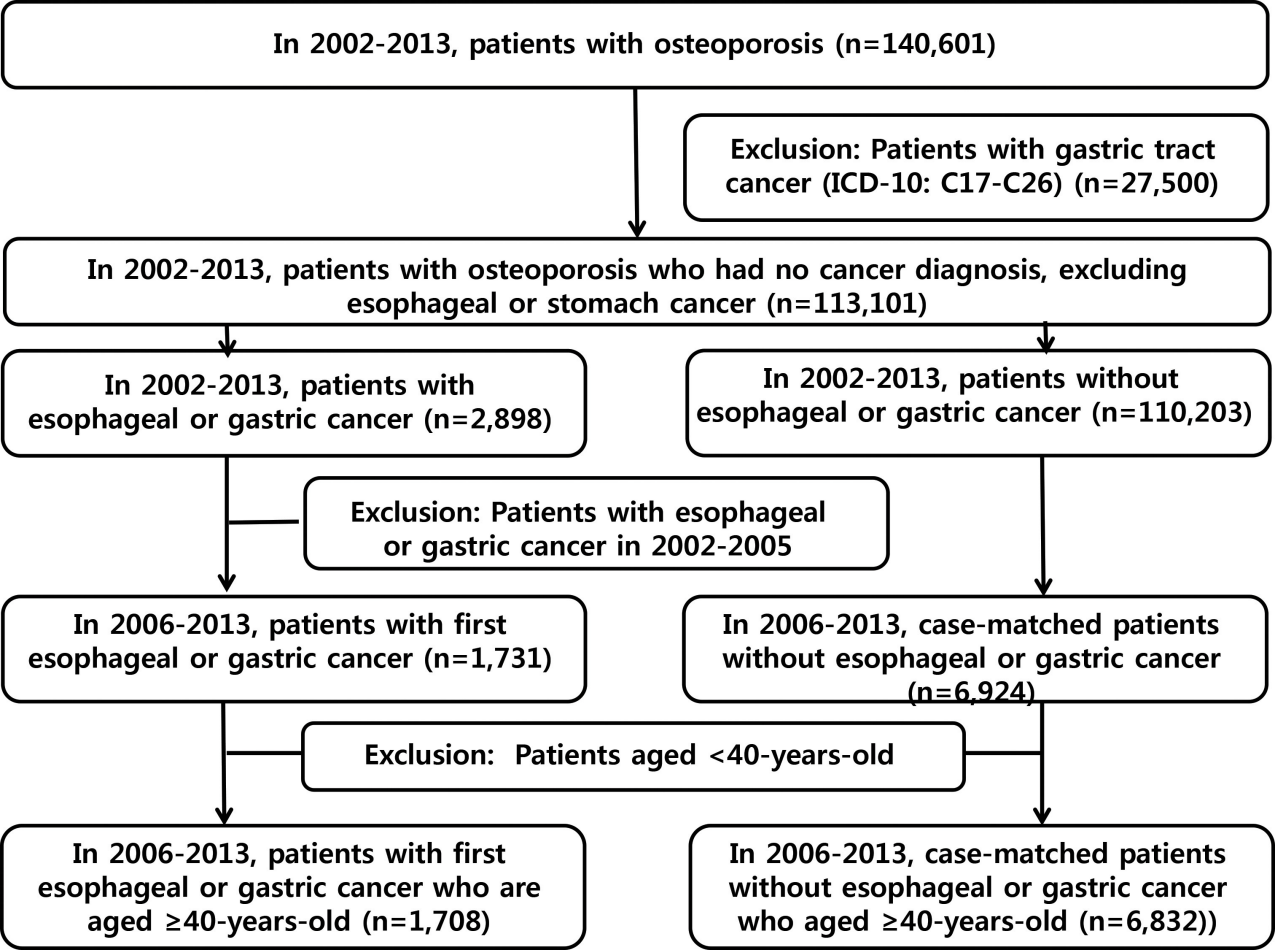
Flow of case and control selection.

We selected 1,708 cases and 6,832 controls (Fig 1). Patient demographics and clinical information are summarized in Table 1. The frequencies of matched variables–such as gender, age, type of insurance, and income level–were the same between case and control groups. But the frequency of concomitant diseases–such as diabetes or rheumatoid arthritis–was slightly different between the groups, showing a trend towards higher co-morbidities in the case group.

**Table 1:** Patient demographics and clinical information for cases and controls.

Characteristics		Case		Control	
		(n = 1,708)		(n = 6,832)	
		n	%	n	%
Sex	Male	570	33.37	2,280	33.37
	Female	1,138	66.63	4,552	66.63
Age (years)^a^	45 to 64	978	57.26	3,912	57.26
	≥65	730	42.74	2920	42.74
Type of insurance	NHI district subscriber	247	14.46	988	14.46
	NHI employee subscriber	1,284	75.18	5,136	75.18
	Medical aid	177	10.36	708	10.36
Income level according to type of insurance^b^	Mean (SD)	7.49	(2.40)	7.50	(2.40)
Comorbidity^c^	Diabetes mellitus without complication^d^	382	22.37	1,189	17.4
	Diabetes mellitus with complication^e^	158	9.25	530	7.76
	Rheumatoid arthritis^f^	120	7.03	353	5.17
	Paget disease of bone^g^	0	0	1	0.01

NHI, national health insurance

^a^ Age was categorized into five year intervals in data

^b^ Income level according to type of insurance: 10 groups for both NHI district subscriber and NHI employee subscriber, and one group as medical aid: total of 21 groups

^c^ Comorbidity was presented for 1–4 years prior to the index date

^d^ E100, E101, E106, E108, E109, E110, E111, E116, E118, E119, E120, E121, E126, E128, E129, E130, E131, E136, E138, E139, E140, E141, E146, E148, E149

^e^ E102, E103, E104, E105, E107, E112, E113, E114, E115, E117, E122, E123, E124, E125, E127, E132, E133, E134, E135, E137, E142, E143, E144, E145, E147

^f^ M05, M06

^g^ M88.

Bisphosphonate exposures during the 2 to 4 years prior to the index date (Period A) and the 1 to 2 years prior to the index date (Period B) are shown in Table 2. During the Period A and B, the absence of bisphosphonate exposure in both periods (non-users) was 82.1% and 83.9%, while the presence of bisphosphonate exposure in both periods (past and recent users) was 6.1% and 5.1%, in the case group and control group, respectively. The ORs (95% CIs) of recent users, past users, and past and recent users of bisphosphonates, after adjusting for concomitant diseases with non-users as a reference, were 1.18 (0.93 to 1.51), 1.04 (0.83 to 1.29) and 1.25 (0.98 to 1.58) (Table 2).

**Table 2:** Association between oral bisphosphonate exposure and esophageal or gastric cancer.

Exposure type	Bisphosphonate prescription		N (%)		OR (95% CI)	
	Period A ^a^	Period B ^b^	Case (n = 1,708)	Control (n = 6,832)	Unadjusted	Adjusted ^c^
Non-user	X	X	1,402 (82.1)	5,730 (83.9)	1	1
Recent user	X	O	92 (5.4)	321 (4.7)	1.18 (0.93–1.51)	1.18 (0.93–1.51)
Past user	O	X	110 (6.4)	436 (6.4)	1.04 (0.84–1.30)	1.04 (0.83–1.29)
Past and recent user	O	O	104 (6.1)	345 (5.1)	1.26 (0.99–1.59)	1.25 (0.98–1.58)^d^

CI, confidence interval; OR, odds ratio

^a^ Period A: 2 to 4 years prior to the index date

^b^ Period B: 1 to 2 years prior to the index date

^c^ Adjusted for diabetes mellitus without complications, diabetes mellitus with complications, rheumatoid arthritis, and Paget disease of bone

^d^ P-value = 0.068.

The ORs (95% CIs) for the CDE level for 3 years (1 to 4 years; Period A+B) prior to the index date, after adjusting for concomitant disease, were 1.16 (0.99 to 1.36), 1.13 (0.80 to 1.59), 0.89 (0.50 to 1.60), and 1.24 (0.66 to 2.33) for 0%<CDE<25%, 25%≤CDE<50%, 50%≤CDE<75%, and 75%≤CDE≤100%, respectively, with a reference of bisphosphonate non-exposure group. The results for 1 to 2 years (only Period A) prior to the index date were similar in trend to the 1 to 4 years results, without statistical significance (Table 3).

**Table 3:** Association between oral bisphosphonate exposure and esophageal or gastric cancer according to CDE.

Bisphosphonate exposure level	N (%)		OR (95% CI)	
(CDE, %)	Case (n = 1,708)	Control (n = 6,832)	Unadjusted	Adjusted ^c^
3 years (1 to 4 years; Period A+B) prior to the index date ^a^				
0	1,402 (82.1)	5,730 (83.9)	1	1
0<CDE<25	235 (13.8)	834 (12.2)	1.16 (0.99–1.36)	1.16 (0.99–1.36)^d^
25≤CDE<50	44 (2.3)	161 (2.4)	1.13 (0.80–1.60)	1.13 (0.80–1.59)
50≤CDE<75	14 (0.8)	64 (0.9)	0.91 (0.51–1.63)	0.89 (0.50–1.60)
75≤CDE≤100	13 (0.8)	43 (0.6)	1.25 (0.67–2.35)	1.24 (0.66–2.33)
1 year (1 to 2 years; Period A) prior to the index date ^b^				
0	1,513 (88.6)	6,167 (90.3)	1	1
0<CDE<25	97 (5.7)	333 (4.9)	1.20 (0.95–1.52)	1.19 (0.94–1.50)
25≤CDE<50	43 (2.5)	139 (2.0)	1.28 (0.90–1.82)	1.30 (0.91–1.85)
50≤CDE<75	19 (1.1)	85 (1.2)	0.92 (0.56–1.53)	0.93 (0.56–1.54)
75≤CDE≤100	36 (2.1)	108 (1.6)	1.38 (0.94–2.03)	1.36 (0.93–2.01)^e^

CI, confidence interval; OR, odds ratio.

CDE, Cumulative Duration of Exposure, calculated as the duration (percentage) of the total prescription days in years during the 1 to 4 year period (3 years) or the 1 to 2 year period (1 year) prior to the index date

^a^ 3 years (1 to 4 year; Period A (1 to 2 years prior to the index date) + Period B (2 to 4 years prior to the index date)) prior to the index date.

^b^ 1 year (Period A; 1 to 2 years) prior to the index date.

^c^ Adjusted for diabetes mellitus without complications, diabetes mellitus with complications, rheumatoid arthritis, and Paget disease of bone

^d^ P-value = 0.073

^e^ P-value = 0.116.

Table 4 shows the sensitivity analysis results and the overall results were similar to the base case analysis results. When bisphosphonate exposure included the injection drug formulation type as well as the oral, no statistical significance was shown. When gastric or esophageal cancers were investigated alone, neither group had a statistical association. When cases were limited to only those involving hospitalization or surgical operation or anticancer drug use, significant associations were still not observed. In addition, when we did not exclude any gastrointestinal tract cancer from the cohort population, a past and recent user group showed the significant association (OR = 1.25, 95% CIs = 1.04–1.50), but the 75%≤CDE≤100% did not have a significant statistical association (OR = 1.24, 95% CIs = 0.72–2.12).

**Table 4:** Sensitivity analyses of association between bisphosphonate exposure and esophageal or gastric cancer.

Prescription of Bisphosphonate	Adjusted odds Ratio ^a^	CDE ^b^	Adjusted odds Ratio ^a^
Bisphosphonate (INJ + Oral)			
		0	1
Non-user	1	0<-<25%	1.10 (0.93–1.29)
Recent user	1.20 (0.94–1.52)	25≤-<50%	1.13 (0.81–1.58)
Past user	0.98 (0.79–1.22)	50≤-<75%	1.03 (0.59–1.79)
Past and recent user	1.19 (0.94–1.50)	75≤-≤100%	1.34 (0.74–2.43)
Definition of case: Esophageal cancer only (Case n = 230, Control n = 920)			
		0	1
Non-user	1	0<-<25%	1.10 (0.71–1.69)
Recent user	1.46 (0.78–2.75)	25≤-<50%	0.12 (0.03–1.49)
Past user	0.74 (0.38–1.44)	50≤-<75%	0.78 (0.09–6.81)
Past and recent user	0.81 (0.36–1.80)	75≤-≤100%	1.47 (0.15–14.22)
Definition of case: Gastric cancer only (Case n = 1,505, Control n = 6,020)			
		0	1
Non-user	1	0<-<25%	1.16 (0.98–1.38) ^c^
Recent user	1.17 (0.90–1.52)	25≤-<50%	1.25 (0.88–1.78)
Past user	1.06 (0.84–1.34)	50≤-<75%	0.90 (0.49–1.66)
Past and recent user	1.29 (1.00–1.65) ^c^	75≤-≤100%	1.22 (0.63–2.35)
Definition of case: Esophageal or gastric cancer with hospitalization (Case n = 1,258, Control n = 5,032)			
		0	1
Non-user	1	0<-<25%	1.24 (1.03–1.49) ^c^
Recent user	1.31 (0.99–1.73) ^c^	25≤-<50%	1.09 (0.73–1.61)
Past user	1.05 (0.82–1.36)	50≤-<75%	0.78 (0.38–1.61)
Past and recent user	1.24 (0.94–1.63)	75≤-≤100%	1.01 (0.46–2.22)
Definition of case: Esophageal or gastric cancer with surgical operation or anticancer drugs (Case n = 586, Control n = 2,344)			
		0	1
Non-user	1	0<-<25%	1.16 (0.87–1.54)
Recent user	1.09 (0.70–1.71)	25≤-<50%	1.19 (0.66–2.14)
Past user	1.08 (0.75–1.560)	50≤-<75%	0.85 (0.35–2.08)
Past and recent user	1.17 (0.76–1.80)	75≤-≤100%	-^d^
In case of not excluding gastrointestinal tract cancer (Case n = 2,731, Control n = 10,924)			
		0	1
Non-user	1	0<-<25%	1.14 (1.00–1.29) ^c^
Recent user	1.14 (0.93–1.38)	25≤-<50%	1.13 (0.86–1.48)
Past user	1.05 (0.88–1.25)	50≤-<75%	1.12 (0.75–1.68)
Past and recent user	1.25 (1.04–1.50) ^c^	75≤-≤100%	1.24 (0.72–2.12)

^a^ Adjusted for diabetes mellitus without complications, diabetes mellitus with complications, rheumatoid arthritis, and Paget disease of bone (osteitis deformans)

^b^ Cumulative Duration of Exposure (CDE), calculated as the duration (percentage) of the total prescription days in years during 1 to 4 year prior to index date

^c^ P-value <0.1

^d^ It was not calculated due to no cases.

## Discussion

Based on this population-based, case-control study, bisphosphonate exposure groups did not show an increased risk of esophageal or gastric cancer, even when several different outcome definitions were applied. Also, there was no association between bisphosphonate exposure level and an increased risk of esophageal or gastric cancer. Our results are in line with recent US study that examined cumulative pills of bisphosphonate exposure intensity and upper gastrointestinal cancers[28].

In addition, results on esophageal cancer add information of non-significant dose-response relationship to a recent cohort study that showed no significant association between oral bisphosphonates and esophageal cancer in Korean women with osteoporosis (adjusted hazard ratio 0.87 (0.39–1.98))[29], but lacked information on duration or dose of bisphosphonate exposure. Other studies also reported no increased risk of esophageal cancer in general population[12, 30], osteoporotic patients[31], or Barret’s esophagus patients[32]. Unlike these study findings, Green et al. reported a significant association between long-term (3 years or more) or high intensity (10 or more prescriptions) oral bisphosphonates use and esophageal cancer[13], based on a case-control analysis using two UK primary care databases. Given recent studies of the Asian population, including Korean and Taiwan, there might be a genetic variation involved in esophageal cancer. Besides, based on studies done in the US, or other EU countries that also reported no significant association, other factors in the UK population that were linked to prolonged bisphosphonate use may have increased the risk of esophageal cancer.

For gastric cancer, our analysis showed a marginally significant association among patients who were exposed in both exposure windows. However, analysis according to CDE showed no significant trends to this relationship. Given these conflicting results, we demonstrated that there was no dose-relationship between a longer duration of bisphosphonate exposure and gastric cancer. A study using UK primary care databases demonstrated a significant risk in two or more times of alendronate use, which was limited for only short-term use. This short-term association was interpreted as stopping bisphosphonate might be due to earlier detection of existing cancer, or simply be a spurious chance finding[12]. In several studies, bisphosphonates showed no significant relationship with gastric cancer[13, 30]. On the other hand, a Danish cohort study[31] demonstrated a decreased risk of gastric cancer in the women who used alendronate, with a higher prevalence of receiving upper endoscopy (which can imply a bias due to intensive screening of upper GI adverse effects).

A potential mechanism by which oral bisphosphonates increase cancer risk has been suggested to be due to injury, inflammation, and irritation caused by contact between the bisphosphonate pills and the esophageal or gastric mucosa[33, 34]. Studies on bisphosphonates and upper GI cancers are prone to the problem of protopathic bias, or early detection of upper GI cancer. To avoid this issue, we excluded recent cancers (1 year preceding the index date) from our exposure definition. Further, in pharmacoepidemiologic analysis–especially those involving chronic drug use–time-varying exposure statuses need to be considered[35]. We assessed the exposure status of bisphosphonates using two approaches, exposure time windows and cumulative duration of exposures, to avoid misinterpretation arising from exposure misclassification.

Findings from this study would have a high external validity and generalizability because we used cohort sample data from the National Health Insurance Service- Cohort Sample Database, which covers the entire population in Korea. The database details health, national health insurance claims data, death records, and socio-demographic information, including income-level data.

There are several limitations of this study. First, by the nature of case-control design, selection of appropriate controls with similar characteristics of cases excluding exposure of interest (i.e., bisphosphonate exposure in our study) is a very important factor for avoiding bias. With any retrospective, longitudinal study, unmeasured confounders cannot be absolutely removed. In this study, we aimed to find a comparable control group by matching on sex, age, income level, and insurance type. The baseline characteristics between the two groups did not differ significantly from each other. Furthermore, we adjusted for major co-morbidities showing differences between the two groups.

Second, definition of cancer diagnosis using ICD-10 code recorded in the NHIS database established for reimbursement claims may pose an accuracy issue. In the past, accuracies of cancer diagnoses recorded in the NHIS data source using ICD-10 codes were not good as 78% in the agreement rate between diagnosis codes recorded in NHIS database during 1999 and 2001 and the patients’ medical records for the malignant tumors, reported in one study[36]. But, since 2006 when the policy of extending the health insurance benefit coverage to lower the out-of-pocket money for cancer patients in Korea was implemented, accuracies of diagnoses recorded in the NHIS database became very high[37]. Accordingly, the NHIS database from 2006 to 2013 we used in this study could be considered to be guaranteed accuracies of cancer diagnoses. On the other hand, we carried out sensitivity analyses for the cases defined by esophageal or gastric cancer diagnosis codes together with hospitalization, or surgical operation or anticancer drugs, in order to increase accuracy of case selection. These various sensitivity analyses may overcome a probable lack of accuracy of the claims database and provide the robustness of the study results.

Third, this study examined relatively short-term exposure and latency. Thus, we could not address an increase or decrease in risks associated with longer exposure or latency of bisphosphonate more than 4 years prior to index date. There was a case-control study which followed up for 10 years reporting an increase of esophageal cancer in patients who used bisphosphonates for more than 5 years[13]. Accordingly a further study with availability of expanded data ensuring much longer period of exposure and latency than this study is expected in the future.

Fourth, medication adherence rate in patients enrolled in this study seemed not to be sufficient. This low adherence together with relatively short cumulative drug exposure duration might weaken to find the association of drug-cancer. Further study in a larger number of subjects with higher exposure and higher adherence is necessary to prove more precise findings.

Fifth, we did not measure established confounders as potential risk factors for upper GI cancers such as body mass index(BMI), smoking, salty foods, etc.[38–43] due to lack of information. NHIS database we used in this study did not include individual health behaviors and daily life habits. Thus, we matched sex instead of smoking, and matched age, sex, and income level instead of BMI in selecting controls. But study results did not show any different direction by these factors. Accordingly, BMI and smoking seemed not to be significant confounders within the database in this study even though they would be risk factors for gastric cancers. In a previous study by Green et al. conducted in the UK, bisphosphonate-associated risk also did not vary materially between groups of patients categorized by age, sex, smoking status, alcohol intake as well[13].

In conclusion, this study did not prove a significant association between bisphosphonates and upper gastrointestinal cancer in an Asian population-based, real-world setting. But, the marginally increased risk in some exposure groups was shown even though there was no statistical significance due to a limited number of subjects and relatively shorter period of follow up.
